# Estimating the Effect of a Bovine Viral Diarrhea Virus Control Program: An Empirical Study on the Performance of Dutch Dairy Herds

**DOI:** 10.3389/fvets.2022.892928

**Published:** 2022-07-07

**Authors:** Xiaomei Yue, Jingyi Wu, Mariska van der Voort, Wilma Steeneveld, Henk Hogeveen

**Affiliations:** ^1^Business Economics Group, Department of Social Sciences, Wageningen University, Wageningen, Netherlands; ^2^Department of Population Health Sciences, Faculty of Veterinary Medicine, Utrecht University, Utrecht, Netherlands

**Keywords:** dairy, bovine viral diarrhea virus, control program, propensity score matching, Difference-in-Differences, economic

## Abstract

More and more European countries have implemented a bovine viral diarrhea virus (BVDV) control program. The economic effects of such programs have been evaluated in simulations, but empirical studies are lacking, especially in the final stage of the program. We investigated the economic (gross margin) and production effects (milk yield, somatic cell count, and calving interval) of the herds obtaining BVDV-free certification based on longitudinal annual accounting and herd performance data from Dutch dairy herds between 2014 and 2019, the final stages of the Dutch national BVDV-free program. This study was designed as a case-control study: two types of case herds were defined for two analyses. The case herds in the first analysis are herds where the BVDV status changed from “BVDV not free” to “BVDV free” during the study period. The not-free status refers to a herd that participated in the BVDV-free program but had not yet obtained the BVDV-free certification. In the second analysis, the case herds started participating in the Dutch BVDV-free program during the study period and obtained the BVDV-free certification. Control herds in both analyses were BVDV-free during the entire study period. Potential bias between the covariates of the two herd groups was reduced by matching case and control herds using the propensity score matching method. To compare the differences between case and control herds before and after BVDV-free certification, we used the time-varying Difference-in-Differences estimation (DID) methodology. The results indicate that there was no significant change in milk yield, somatic cell count, calving interval, and gross margin upon BVDV-free certification. There are several possible explanations for the non-significant effects observed in our study, such as the final stage of the BVDV control program, not knowing the true BVDV infection situation in case herds and not knowing if control measures were implemented in case herds prior to participating in the BVDV-free program. In our study, the effects of BVDV-free certification might have been underestimated, given that the Dutch BVDV control program became mandatory during the study period, and some of the case herds might have never experienced any BVDV infection. The results of this study suggest that in the final stage of the BVDV control program, the program may no longer have a clear benefit to the herd performance of participating dairy herds. When designing national programs to eradicate BVDV, it is therefore important to include incentives for such farms to motivate them to join the program.

## Introduction

Bovine viral diarrhea (BVD) is a contagious cattle disease, reported in 88 countries worldwide (1, 2). Infections with bovine viral diarrhea virus (BVDV) cause reproductive disorders (e.g., infertility, early embryonic deaths, abortion, prolonged calving interval) and reduce productivity (e.g., reduced milk yield, increased premature culling and mortality among calves and cows), resulting in poor herd and economic performance (3–5). Direct monetary losses due to BVDV range from 0.45 to 604.13 euros per animal per year (1).

Four Scandinavian countries (Norway, Sweden, Finland, and Denmark) launched national BVDV eradication programs in the early 1990s, and entered the final stage of eradication 10 years later (6, 7). This successful experience inspired other European countries (e.g., Switzerland, Germany, Ireland, UK, and France) to implement voluntary or compulsory BVDV control programs (8–12). In the Netherlands, a voluntary BVDV control program, the so-called “BVDV-free” program, was launched in 1997 (13). In 2014, 34% of the Dutch dairy herds had a BVDV-free or BVDV-unsuspected status (14) and this percentage increased to 73% in 2019 (15). To further eradicate BVDV, the national mandatory BVDV control program started from April 1, 2018 (16), which marked the final stage of the BVDV program. As of the first quarter of 2021, already 84% of Dutch dairy farms had a BVDV-free or BVDV-unsuspected status, up from 65% in the first quarter of 2018 (17, 18).

There is a growing body of literature that estimates the effects of the implementation of BVDV control measures on production [e.g., milk yield, somatic cell count (SCC), calving interval] of dairy farms. Existing empirical studies have found that the positive effects of BVDV eradication on production are limited. For instance, Tschopp et al. (19) found no significant difference in milk yield before and after BVDV eradication in Swiss dairy farms, but reported a slight decrease in bulk milk SCC after the herds were declared free of BVDV infection. Contrary, Berends et al. (20) found no significant changes in bulk milk SCC after the Dutch dairy herds were certified as BVDV-free. Similarly, no significant difference in calving interval was observed between BVDV-free herds and herds with at least one persistently infected (PI) animal in Austria (21). Berends et al. (20) was the most recent Dutch study that empirically evaluated the effects of becoming BVDV free. How these may have changed following the introduction of the (mandatory) Dutch BVDV control programs remains unexplored. Also, previous research on the effects of BVDV control programs has mostly focused on the early stages of the program [e.g., (22–24)] while it is unclear whether there are production and/or economic benefits for herds certified as BVDV-free in the final stages of the programs.

A recent review summarized the economic consequences of BVDV prevention and mitigation activities in twelve countries worldwide (25). Control programs were economically justified in only four countries (i.e., Norway, Ireland, France, and Switzerland), based on simulation models (23, 26–28). In the Netherlands, Santman-Berends et al. (29) simulated the economic effects of different BVDV control scenarios. Besides these published simulation studies, to our knowledge, no empirical studies exist that are based on accounting data. Therefore, the economic effects of the Dutch BVDV-free program are still unknown.

The objective of our study was to empirically investigate the economic (gross margin) and production effects (SCC, calving interval, and milk yield) of the herds obtaining BVDV-free certification in the final stage of a national BVDV control program. The analysis will be based on recent longitudinal annual accounting and herd performance data.

## Materials and Methods

### Data Collection

Longitudinal herd-level data on herd characteristics, herd performance, accounting and BVDV status were provided by Dirksen Management Support (DMS, Beusichem, the Netherlands). The data represented 1,828 yearly observations of 456 anonymized Dutch dairy herds cooperating with DMS over the years 2011–2019. Herd characteristics included land use and herd size. Herd performance consisted of data on total milk production, milk fat and protein percentage, calving interval, non-return rate, age of culled cows, number of culled cows, number of inseminations, etc. The annual accounting data involved total revenues (consisting of milk revenues and calf and cattle revenues), fixed and variable costs (e.g., feed costs, fertilizer costs, animal health costs), and gross margin (i.e., total revenues minus total variable costs). For each year, the BVD status of the herd was categorized as either “BVDV free,” “BVDV not free,” or “not-participating.” BVDV-free means that the herd has participated in the BVDV control program and obtained BVDV-free certification, while BVDV not free means that the herd has participated in the control program but had not yet obtained the BVDV-free certification. Not-participating means that the herd does not participate in the BVDV-free program.

### Data Editing

Three new variables were generated: farm intensity (no. of milking cows/hectare/year), milk yield (kg/cow/year), and gross margin (euros/kg milk/year). The data were cleaned as follows. Firstly, 482 observations with missing data on BVDV status or with meaningless values for any of the other variables (e.g., SCC = 0, calving interval = 0) were removed. Secondly, only herds with data for at least two consecutive years were included, resulting in the exclusion of 68 observations from the data set. Thirdly, distributions of each variable were visually checked and 21 observations with extreme values (i.e., farm intensity >5 cows/hectare, herd size >400 cows, land use >200 hectares) were excluded. Fourthly, as there were only five observations in 2011 and 2012, the observations in these years were excluded.

After these cleaning steps, the data set consisted of 1,252 observations from a total of 270 herds. Among the 270 herds, 21 herds changed from “BVDV not free” to “BVDV free,” 51 herds changed from “not participating” to “BVDV free” and 156 herds remained “BVDV free” during the study period. The other 48 herds either remained “BVDV not free,” remained “not participating,” changed from “not participating” to “BVDV not free,” or changed from “BVDV free” to “BVDV not free.” We focused our study on two types of herds obtaining the BVDV-free certification: (i) from “BVDV not free” to “BVDV free” (i.e., first analysis); (ii) from “not participating” to “BVDV free” (i.e., second analysis).

In the first analysis production and economic parameters of herds that changed from “BVDV not free” to “BVDV free” (case herds) were compared with herds that remained BVDV free for the entire study period (control herds). Case herds obtained the BVDV-free certification in five different years: 2015–2019. Therefore, five sub-data sets were created: one for each year in which a case herd became “BVDV free.” Each sub-data set included data of the year where the case herds obtained the BVDV-free certification (i.e., year 0), data of the preceding year (year−1, with “BVDV not free” status) and data of the following year (year 1, with “BVDV free” status). For each sub-dataset, the respective for the same 3 years from the control herds were added. Because data on the year 2020 was not available, the sub-data set for herds changed BVDV status in 2019 only included observations of 2 years (2018 and 2019). Consequently, the five sub-data sets included data between 2014 and 2019. The five sub-data sets for the first analysis contained 310, 291, 306, 258, and 180 observations from 100, 98, 103, 87, and 90 dairy herds, respectively.

For the second analysis, case herds were defined as herds changed from “not participating” to “BVDV free.” The procedures to generate sub-data sets in this analysis were similar to the first analysis, resulting in five sub-data sets containing 310, 323, 322, 270, and 180 observations from 104, 108, 108, 92, and 90 dairy herds, respectively. Data editing was conducted in R version 4.0.5 (30).

### Matching Case and Control Herds

The fact that studied case and control herds were not randomly assigned may have led to selection bias (31). To reduce this potential bias, for both analyses case and control herds were matched using the propensity score matching (PSM) method (32–34). Case and control herds were matched based on herd size, farm intensity, and milk yield in the year that the case herds were certified as BVDV-free for the estimation of the effect of becoming BVDV free on calving interval and gross margin. For the analyses on milk yield and SCC, herd size and farm intensity were used to match case and control herds. The PSM procedure was performed with the psmatch2 module (35) in STATA version 15 (36). The propensity scores were estimated through a logit regression of the BVDV-free certification on the selected covariates (32). Different matching algorithms for matching on the propensity score (nearest neighbor matching without replacement, 1:1, 1:2, 1:3, 1:4 nearest neighbor matching with 0.01 caliper width, kernel matching) were examined to determine the most suitable matching method for each sub-data set. The matching performance of different algorithms was assessed by comparing the number of matched groups and the standardized percentage bias (generally <5%) after matching with the post-estimation command pstest (37–39). The selected PSM matching algorithms for each sub-data set in the first analysis (case herds defined as herds changing from “BVDV not free” to “BVDV free”) are listed in Table 1. Three matching algorithms were selected: 1:4 nearest neighbor matching with 0.01 caliper width (control herds were matched with replacement), 1:2 nearest neighbor matching with 0.01 calip width, and Kernel matching. The balancing of the covariates in all sub-data sets before and after matching are presented in the Supplementary Tables 1, 2. Compared with the unmatched results, the standardized percentage bias of most covariates was reduced after matching. This proves the effectiveness of PSM in reducing the confounding effects between the case and control herds in the first analysis and prepares for further statistical analysis. The matched sub-data sets in the first analysis were merged into two final panel data sets (Table 2): one data set for analyzing calving interval and gross margin (i.e., data set 1.1, including 116 herds); and one data set for analyzing milk yield and SCC (i.e., data set 2.1, including 114 herds).

**Table 1:** Selected matching algorithms for five sub-data sets of the first (case herds changed from “BVDV not free” to “BVDV free”) and second analyses (case herds changed from “not participating” to “BVDV free”) for calving interval, gross margin milk yield, and somatic cell count (SCC).

**Sub-data set**	**Selected matching method for analysis of calving interval and gross margin**	**Selected matching method for analysis of milk yield and SCC**
**First analysis (case herds changed from “BVDV not free” to “BVDV free”)**		
2015	1:2 nearest neighbor matching with 0.01 caliper width	Kernel matching
2016	Kernel matching	1:4 nearest neighbor matching with 0.01 caliper width
2017	Kernel matching	Kernel matching
2018	Kernel matching	1:4 nearest neighbor matching with 0.01 caliper width
2019	1:4 nearest neighbor matching with 0.01 caliper width	1:4 nearest neighbor matching with 0.01 caliper width
**Second analysis (case herds changed from “not participating” to “BVDV free”)**		
2015	1:2 nearest neighbor matching with 0.01 caliper width	Kernel matching
2016	1:2 nearest neighbor matching with 0.01 caliper width	Kernel matching
2017	Kernel matching	Kernel matching
2018	1:2 nearest neighbor matching with 0.01 caliper width	Kernel matching
2019	Kernel matching	1:2 nearest neighbor matching with 0.01 caliper width

**Table 2:** Summary of the matched sub-data sets using the property score matching method and the merged final four data sets in the first^a^ and second^b^ analyses.

**Analyzed parameters**	**Sub-data sets**	**Data set 1.1 (first analysis)**		**Data set 1.2 (second analysis)**	
		**No. of case herds (No. of obs.)**	**No. of control herds (No. of obs.)**	**No. of case herds (No. of obs.)**	**No. of control herds (No. of obs.)**
Calving interval, gross margin	2015	2 (6)	3 (9)	7 (21)	12 (36)
	2016	3 (7)	26 (78)	12 (36)	21 (63)
	2017	7 (20)	79 (237)	12 (36)	86 (258)
	2018	1 (3)	64 (192)	6 (15)	12 (36)
	2019	4 (8)	9 (18)	4 (8)	44 (88)
	Total^*c*^	17 (44)	99 (534)	41 (116)	101 (481)
**Analyzed parameters**	**Sub-data sets**	**Data set 2.1 (first analysis)**		**Data set 2.2 (second analysis)**	
		**No. of case herds (No. of obs.)**	**No. of control herds (No. of obs.)**	**No. of case herds (No. of obs.)**	**No. of control herds (No. of obs.)**
Milk yield, somatic cell count	2015	3 (9)	37 (111)	7 (21)	66 (198)
	2016	4 (10)	15 (45)	14 (42)	76 (228)
	2017	8 (23)	78 (234)	12 (36)	80 (240)
	2018	1 (3)	4 (12)	6 (15)	63 (189)
	2019	4 (8)	13 (26)	4 (8)	8 (16)
	Total	20 (53)	94 (428)	43 (122)	109 (871)

a *First analysis = case herds changed from “BVDV not free” to “BVDV free.” The not-free status refers to a herd that participated in the BVDV-free program but had not yet obtained the BVDV-free certification*.

b *Second analysis = case herds changed from “not participating” to “BVDV free”*.

c *The final data set is panel data, so duplicate records in different sub-data set were removed*.

For the second analysis (case herds defined as herds changing from “not participating” to “BVDV free”), the same PSM procedure was performed as for the first analysis. The selected PSM matching algorithms for each sub-data set in the second analysis are also listed in Table 1. The balancing of the covariates in all sub-data sets before and after matching are presented in the Supplementary Tables 3, 4. The effectiveness of PSM in reducing the confounding effects between the case and control herds in the second analysis was also proved. Two final panel data sets were also prepared for the second analysis (Table 2): data set 1.2 including 142 herds and data set 2.2 including 152 herds.

### Data Analysis

**Figure 1:**
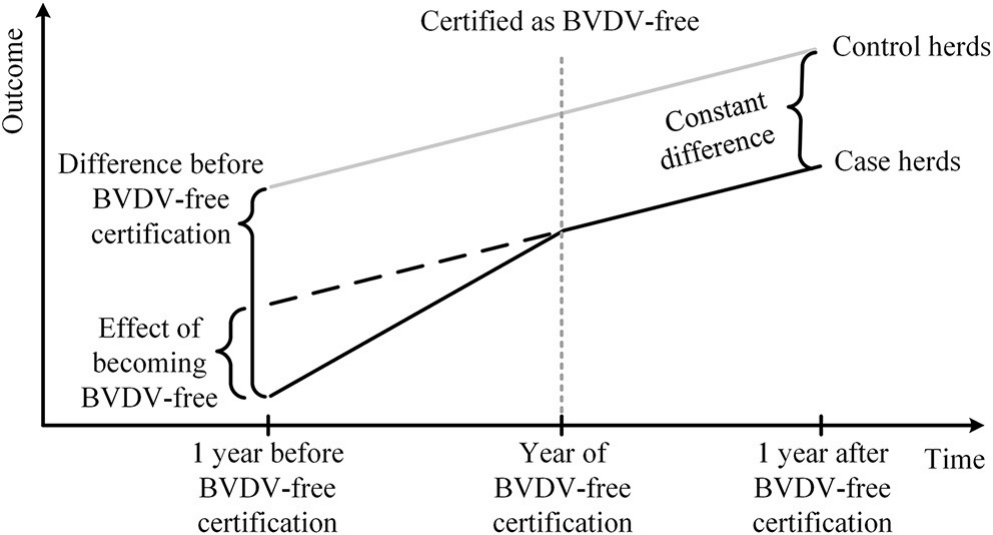
Schematic diagram of using the Difference-in-Differences estimation methodology to analyze the effect of BVDV-free certification on Dutch dairy farms.

To estimate the effect of BVDV-free certification, economic and production performance was compared between case and control herds before and after BVDV-free certification using Difference-in-Differences (DID) estimation methodology, which is commonly used to evaluate the causal effects of an intervention (40–42). Figure 1 illustrates the DID estimation methodology for this study. The constant difference in outcome (i.e., milk yield, SCC, calving interval, and gross margin) between the case and control herds after BVDV-free certification was computed, as well as the difference in outcome between the two groups before the BVDV-free certification. Subsequently, the effect of BVDV-free certification was estimated by subtracting the constant difference from the difference between the two groups before certification.

The time-varying DID estimation methodology, applied to the situation where a program is implemented in multiple time periods, was used in this study (42). The DID estimation methodology was performed in the four panel data sets, with duplicate observations dropped prior to estimation. The regression set-up is as follows (41):


(1) $$\begin{array}{l} \begin{array}{c} ln{\left(Y\right)}_{it}=\alpha +\beta D_{it}\;+\;\delta X_{it}\;+\;\mu_{i}\;+\;\rho_{t}\;+\;\varepsilon_{it,} \end{array}\\ \;\begin{array}{c} i\;=1,\ldots ,n;t\;=2014,\ldots ,2019 \end{array} \end{array}$$

where *Y*_*it*_ represents indicators of calving interval, milk yield, SCC, and gross margin of herd *i* in year *t*. The four analyzed parameters have been natural log-transformed because they failed the normal distribution test. The variable of interest is *D*_*it*_, is a dummy variable, it equals 1 for the “BVDV not free” status in the first analysis and the “not participating” status in the second analysis and equals 0 when the status is BVDV free. The coefficient β indicates the effect of BVDV-free certification on the outcome parameters. *X*_*it*_ refers to the time-varying herd-level control variables, including continuous variables on herd size and milk yield and the categorical variable farm intensity. Farm intensity consists of three categories: small (*n* ≤ 1.6 cows/ha/year, reference category), medium (1.6 < *n* ≤ 2.6 cows/ha/year), and large (2.6 < *n* ≤ 5 cows/ha/year). The categories of farm intensity were defined by the histogram and heterogeneity trend over intervals. Particularly, the independent variable milk yield was excluded from the SCC and milk yield regression analyses, since this control variable could be expected to act as intervener (43). μ_*i*_, ρ_*t*_ are vectors of herd and year dummy variables that account for herd and year fixed effects, and ε_*it*_ is the error term. The DID estimation methodology was performed with the xtreg command (44) in STATA version 15 (36).

## Results

Table 3A provides the descriptive statistics for the variables used to analyze the performance of herds changing from “BVDV not free” to “BVDV free” (first analysis). Data from variables used to match the case and control herds, as well as data for the four dependent variables (i.e., gross margin, calving interval, milk yield and SCC) are presented. For both, the case herds and the control herds, an increasing trend of gross margin can be seen. However, the improvement of the performance over the years in the control herds (increasing trend in milk yield and decreasing trend in calving interval and SCC) does not apply to the case herds. For instance the mean milk yield of control herds increased from 8,860 to 9,060 kg/cow/year over years, while in the case herds the mean milk yield increased from 8,270 to 8.320 kg/cow/year when the herd was certified as BVDV free and then decreased to 8,230 kg/cow/year the following year.

**Table 3A:** The descriptive statistics of the farm structure variables of studied Dutch dairy herds (herd size, land use and farm intensity from dataset 1.1) and performance variables (gross margin and calving interval from dataset 1.1 and milk yield and SCC form dataset 2.1) as used to analyze the change in performance for herds that changed from “BVDV not free” to “BVDV free” (first analysis^a^).

**Variable**	**First analysis (case herds changed from “BVDV not free” to “BVDV free”)**					
	**Case herds**			**Control herds**		
	**Annual BVDV status**			**Years matched to case herds**		
	**Year−1 (not free)**	**Year 0 (free)**	**Year 1 (free)**	**Year−1 (free)**	**Year 0 (free)**	**Year 1 (free)**
Herd size, *n* cows	118 (43)	117 (41)	107 (37)	120 (40)	118 (41)	116 (39)
Land use, ha	55 (18)	55 (17)	55 (16)	56 (19)	56 (19)	57 (18)
Farm intensity, *n* cows/ha/year	2.18 (0.56)	2.17 (0.47)	1.90 (0.29)	2.23 (0.58)	2.14 (0.51)	2.10 (0.49)
Calving interval, days	406 (23)	404 (18)	413 (29)	407 (22)	406 (19)	404 (17)
Gross margin, euro/kg milk	0.238 (0.052)	0.264 (0.056)	0.276 (0.053)	0.260 (0.055)	0.289 (0.051)	0.296 (0.044)
Milk yield, kg/cow/year	8,270 (1,240)	8,320 (1,240)	8,230 (1,450)	8,860 (830)	8,990 (888)	9,060 (885)
SCC, 1,000 cells/mL	193 (80)	170 (58)	175 (61)	171 (52)	161 (47)	156 (48)

In Table 3B, the descriptive statistics for the variables used to analyze the performance of herds changing from “not participating” to “BVDV free” are provided (second analysis). For both, the case herds and the control herds, an improvement of the performance can be seen. Through the years, there is an increasing trend of the gross margin, milk yield per cow and a decreasing trend for SCC. No trend could be seen for calving interval. Complete descriptive statistics for all variables in the four data sets are provided in Supplementary Table 5.

**Table 3B:** The descriptive statistics of the farm structure variables of studied Dutch dairy herds (herd size, land use and farm intensity from dataset 1.2) and performance variables (gross margin and calving interval from dataset 1.2 and milk yield and SCC form dataset 2.2) as used to analyze the change in performance for herds that changed from “BVDV not free” to “BVDV free” (second analysis^b^).

**Variable**	**Data set 1.2 (second analysis, case herds changed from “not participating” to “BVDV free”)**					
	**Case herds**			**Control herds**		
	**Annual BVDV status**			**Years matched to case herds**		
	**Year−1 (not participating)**	**Year 0 (free)**	**Year 1 (free)**	**Year−1 (free)**	**Year 0 (free)**	**Year 1 (free)**
Herd size, n cows	110 (40)	112 (40)	107 (34)	118 (45)	118 (46)	111 (40)
Land use, ha	55 (20)	56 (20)	56 (17)	56 (21)	57 (21)	57 (21)
Farm intensity, *n* cows/ha/year	2.05 (0.46)	2.03 (0.33)	1.95 (0.29)	2.15 (0.48)	2.09 (0.43)	1.99 (0.40)
Calving interval, days	403 (20)	403 (18)	399 (15)	405 (21)	406 (21)	406 (22)
Gross margin, euro/kg milk	0.286 (0.058)	0.288 (0.058)	0.308 (0.052)	0.256 (0.055)	0.291 (0.049)	0.292 (0.044)
Milk yield, kg/cow/year	8,670 (784)	8,790 (808)	8,970 (851)	8,780 (876)	8,910 (866)	9,000 (866)
SCC, 1,000 cells/mL	162 (50)	154 (43)	150 (43)	167 (52)	161 (50)	158 (50)

a *First Analysis = case herds changed from “BVDV not free” to “BVDV free.” The not-free status refers to a herd that participated in the BVDV-free program but had not yet obtained the BVDV-free certification*.

b *Second analysis = case herds changed from “not participating” to “BVDV Free”*.

Table 4 presents the results of the first DID analysis to study the effects of BVDV-free certification on milk yield, SCC, calving interval, and gross margin of Dutch dairy herds changed from “BVDV not free” to “BVDV free” between 2014 and 2019., The results in Table 4 did not demonstrate any statistically significant difference between the herds with BVDV-free status and BVDV not free status. Table 5 shows the results of the second DID analysis estimating the effects of BVDV-free certification on milk yield, SCC, calving interval, and gross margin of herds changed from “not participating” to “BVDV free.” Overall, no effects of BVDV-free certification on economic and production performance were observed.

**Table 4:** Summarized results of the first difference-in-differences analysis (case herds changed from “BVDV not free” to “BVDV free”) for the effects on calving interval (days), gross margin (euros/kg milk/year), milk yield (kg/cow/year) and somatic cell count (1,000 cells/mL) of dutch dairy herds bovine viral diarrhea virus (BVDV)-free certification.

**Effects**	**Categories**	**Calving interval**		**Gross margin**		**Milk yield**		**Somatic cell count**	
		**Exponent of estimated coefficient**	**p-value**	**Exponent of estimated coefficient**	**p-value**	**Exponent of estimated coefficient**	**p-value**	**Exponent of estimated coefficient**	**p-value**
Intercept		431	0.00	0.36	0.00	8,455	0.00	200	0.00
BVDV certification status	BVDV-free	Referent							
	Not-free^a^	0.998	0.81	0.968	0.29	0.985	0.34	0.972	0.65
Herd size, *n* cows		1.000	0.52	1.001	0.35	1.000	0.75	0.999	0.72
Farm intensity, *n* cows/ha/year	Small^b^	Referent							
	Medium^b^	1.005	0.25	0.993	0.82	0.984	0.34	0.949	0.27
	Large^b^	0.991	0.27	0.981	0.57	0.978	0.27	0.917	0.13
Milk yield, kg/cow/year		1.000	0.07	1.000	0.15	–	–	–	–
Year	2014	Referent							
	2015	1.000	0.99	0.746	0.00	1.014	0.11	0.905	0.00
	2016	0.990	0.47	0.656	0.00	1.030	0.01	0.953	0.27
	2017	0.992	0.58	0.941	0.23	1.065	0.00	0.880	0.00
	2018	0.984	0.29	0.907	0.06	1.072	0.00	0.823	0.00
	2019	0.978	0.14	0.887	0.03	1.058	0.00	0.774	0.00

a *The not-free status refers to a herd that participated in the BVDV-free program but had not yet obtained the BVDV-free certification*.

b *The farm intensity variable consists of three categories: Small (n ≤ 1.6 Cows/ha/Year), Medium (1.6 < n ≤ 2.6 Cows/ha/Year), and Large (2.6 < n ≤ 5 Cows/ha/Year)*.

**Table 5:** Summarized results of the second difference-in-differences analysis (case herds changed from “not participating” to “BVDV free”) for the effects on calving interval (days), gross margin (euros/kg milk/year), milk yield (kg/cow/year) and somatic cell count (1,000 cells/mL) of Dutch dairy herds bovine viral diarrhea virus (BVDV)-free.

**Effects**	**Categories**	**Calving interval**		**Gross margin**		**Milk yield**		**Somatic cell count**	
		**Exponent of estimated coefficient**	**p-value**	**Exponent of estimated coefficient**	**p-value**	**Exponent of estimated coefficient**	**p-value**	**Exponent of estimated coefficient**	**p-value**
Intercept		384	0.00	0.43	0.00	8,820	0.00	186	0.00
BVDV certification status	BVDV-free	Referent							
	Not participating^a^	0.996	0.51	1.007	0.72	0.994	0.42	0.999	0.96
Herd size, *n* cows		1.000	0.09	0.999	0.18	1.000	0.59	1.000	0.86
Farm intensity, *n* cows/ha/year	Small^b^	Referent							
	Medium^b^	0.998	0.78	1.016	0.46	0.987	0.25	0.924	0.00
	Large^b^	0.990	0.19	0.998	0.93	0.982	0.18	0.887	0.00
Milk yield, kg/cow/year		1.000	0.61	1.000	0.12	–	–	–	–
Year	2014	Referent							
	2015	0.999	0.93	0.759	0.00	1.013	0.08	0.924	0.00
	2016	0.989	0.14	0.689	0.00	1.021	0.02	0.961	0.15
	2017	0.991	0.23	0.979	0.46	1.055	0.00	0.904	0.00
	2018	0.986	0.04	0.933	0.01	1.060	0.00	0.867	0.00
	2019	0.987	0.16	0.924	0.01	1.064	0.00	0.861	0.00

a *The not-participating status refers to a herd that did not participate in the BVDV-free program*.

b *The farm intensity variable consists of three categories: Small (n ≤ 1.6 Cows/ha/Year), Medium (1.6 < n ≤ 2.6 Cows/ha/Year), and Large (2.6 < n ≤ 5 Cows/ha/Year)*.

## Discussion

In this case-control study, the economic (gross margin) and production effects (SCC, calving interval, and milk yield) of BVDV-free certification were investigated using PSM and DID approach. Two analyses were performed on two types of herds obtained the BVDV-free certification. The first analysis was on herds changed BVDV status from “BVDV not free” to “BVDV free,” and the second analysis was on herds changed from “not participating” to “BVDV free.” This study is based on longitudinal annual herd performance and accounting data of 152 Dutch dairy herds from 2014 to 2019. Though small changes were observed in the descriptive statistics in milk yield, SCC and gross margin, the DID results indicated that the four analyzed parameters did not significantly change when herds were certified as BVDV free. In other words, for herds whose status changed from “not participating” to “BVDV-free” or “BVDV not free” to “BVDV free,” no changes were observed in milk yield, SCC, calving interval, and gross margin compared to herds that were BVDV-free during the entire study period.

The current study was designed as a case-control study. In order to estimate the effects of BVDV-free certification on the premise of eliminating potential confounding effects between case and control herds, the PSM-DID approach was used. The DID estimation methodology provides unbiased effect estimates if there is a parallel trend in the analyzed parameters between the case and control herds in the absence of the control program (45). Propensity score matching is commonly used to achieve this parallel trend assumption of DID (46). In this study, different PSM algorithms were conducted in different sub-data sets to determine the most suitable matching method for each sub-data set. The three selected matching methods, 1:2 nearest neighbor matching with 0.01 caliper width, 1:4 nearest neighbor matching with 0.01 caliper width and Kernel matching, while ensuring matching performance, matched as many case and control herds as possible. The smaller standardized percentage bias after matching confirms the efficacy of the matching process. However, observations retained after the matching process are limited. For instance, in the second analysis, 489/1.252 observations were included in the calving interval and gross margin analysis, while 627/1,252 were included in the milk yield and SCC analysis. Future studies should include more observations and herds to further investigate the effects of the BVDV-free program.

To the best of our knowledge, our study is the first to use herd characteristics, herd performance, accounting, and BVDV status data to analyze the effects of the Dutch BVDV-free program. Nevertheless, further empirical studies are suggested considering the nature of the available data in this study. As a retrospective case-control study, the study herds were not randomly selected, which could have introduced selection bias. All dairy herds included in this study cooperated with DMS, and there are farmers' seminars every year to discuss how to improve the farm performance (expert opinion obtained from interview). Therefore, the dairy farmers in this study can be characterized as farmers with an above-average interest in optimizing farm management, who are committed to improving economic performance. In addition, during the study period from 2014 to 2019, the studied herds performed better than the average Dutch dairy herds, with a larger herd size (116 cows, average in the studied herds) and milk yield than the national average [116 vs. 98 cows, and 8,910 vs. 8,733 kg/cow/year, average in the studied herds and (47)]. Therefore, further research including more herds with a higher variety in characteristics (e.g., herd size, cattle breed, region) might result in different results. Furthermore, the covariates used for PSM are limited. Due to data availability issues, only herd size, farm intensity, and milk yield (for milk yield analysis, the first two were used) were selected to match case and control herds. The matching outcome will be more accurate if more covariates are included, such as breeding, region, etc., although this will also result in the exclusion of more herds from the analysis (48).

Our empirical analysis shows no significant change in production and economic performance of dairy herds certified as BVDV free. This result was unexpected, as previous observational studies found an increase in SCC (19, 43) and calving interval (21, 49) and a decrease in milk yield (43, 50) in BVDV-infected herds. On the other hand, other published empirical studies on the effects of the BVDV control program also did not find significant differences in SCC (20), calving interval (21), and milk yield (19) between case and control herds. An important reason for such non-significant findings in these and our study may be the uncertain infection dynamics. Berends et al. (19–21), all mentioned that case herds may have developed immunity prior to the eradication of BVDV, thereby protecting the herd from bigger losses. Information about the infection dynamics of case herds before the eradication of BVDV is often not available in retrospective data, which makes it difficult to detect significant results or to explain non-significant results. Although previous studies have reported negative effects of BVDV infection on gross margin (7, 51, 52), these were all based on normative simulation models. These ex-ante studies can support decisions regarding BVDV control programs and are often conducted before the implementation of such programs. However, for future decision making, it would also be useful to perform ex-post studies once the BVDV control programs are being carried out. Such economic empirical ex-post studies have not been conducted to date, which reflects the need for additional studies in the future.

Three more specific reasons may explain the absence of effects upon BVDV-free certification in the present study. First, before the BVDV-free certification, case herds were either herds not participating in the BVDV-free program or herds participated in the BVDV-free program but had not yet obtained the BVDV-free certification. However, the real BVDV infection situation of case herds before BVDV-free certification cannot be accurately defined without knowing the presence of PI animals (the main reservoir of BVDV and shed a large number of viruses to susceptible animals throughout their lives) and the antibody prevalence. On the one hand, if PI animals present in the case herds before the BVDV-free certification, it is expected that economic and production performance will improve after BVDV-free certification, as the negative impact of BVDV infection has been reported to be larger in herds with PI animals than without (6, 53, 54). On the other hand, if the antibody prevalence of case herds was relatively high before the BVDV-free certification, the changes in economic and production performance are likely to be small. The high antibody prevalence associated with lifelong immunity in the majority of the cows in the herd could protect the herd from serious negative effects (2, 55–58). A possible way to define the infection status of case herds more precisely is to consider the BVDV test results prior to BVDV-free certification, such as the presence of (newborn) PI animals. For example, in studies of Toplak et al. (20, 59), the presence of at least 1 PI animal was set as a necessary condition to define the case herds. Secondly, the case herds in our study were the “tail herds,” meaning that they were the latest herds that started to participate in the BVDV-free program. These herds may have started so late because of having not many BVDV-related problems compared to those who participated in the program two decades ago. Or the case herds (applicable for the second analysis) may have already implemented some control measures before participating in the program. Finally, this study was based on annual herd performance and accounting data, and only 3 yearly observations per herd were included. If more frequent data with more observations will be available, it will be possible to analyze economic and production performance before and after the BVDV-free certification more precisely.

In our study, no differences in gross margin were found when the herds obtained the BVDV-free certification. In addition to the possible general reasons discussed above, another potential explanation for the economic indicator is that the costs of BVDV-free certification have leveled out the potential positive economic consequences of being BVDV-free. In both the voluntary (prior to 2018) or mandatory (after 2018) BVDV control program in the Netherlands, dairy farmers pay all costs related to BVDV control, such as virus testing costs of newborn calves for 10 months in the intake phase, the virus or antibody testing costs in the monitoring phase, and the costs of removing PI animals [if any, van Duijn et al. (60)]. Therefore, these animal health costs may balance the positive economic consequences of BVDV-free certification (e.g., increased milk sales, decreased premature culling, etc.). In addition, gross margin is a useful indication to compare the economic performance of different herds, but it can be strongly influenced by management factors other than BVDV-free certification. The management factors can be partly triggered by policy alterations (61). During the study period, the milk quota system was abolished in 2015, and phosphate regulation was implemented in 2017 (62–64). As a consequence, different study herds may have taken different strategic management decisions, thereby affecting gross margin (61, 65). So while herd and year effects were included in the DID model, it is difficult to eliminate the impact of management changes on gross margin due to the large heterogeneity of farm management decisions.

The effects of the Dutch BVDV-free program may have been underestimated due to the study period. During the study period, the BVDV control campaign in the Netherlands came to the tail end with the implementation of the compulsory schemes in 2018. On the one hand, the voluntary BVDV control started in 1997 has made effective progress before the study period. A prevalence study from GD animal health in 2013/2014 showed that only 14% of dairy farms had a recent BVDV circulation (14), and this percentage dropped to 8.7% in 2015/2016 (17). This drop during the study period suggests that the case herd may not have the BVDV circulation before certifying as BVDV free. On the other hand, BVDV control became mandatory in Dutch dairy herds during the study period, so even the dairy herds that had never experienced BVD related issues were obliged to participate in the program and to achieve the official free or unsuspected status. Such dairy herds may also be included in the case herds, resulting in an underestimation of the effectiveness of BVDV control.

The results of this study suggest that in the final stage of the BVDV control program, the program may no longer have a clear benefit to the herd performance of participating dairy herds. Most likely, herds that benefit from BVDV-free certification, for instance because of reduced production losses or trading benefits, did enter the program in an earlier stage. When a BVDV program is designed to eradicate BVDV at the national level, it is therefore important to provide incentives for herds without clear benefits to become BVDV free. If such incentives are not provided, a program may not be successful in eradicating BVDV.

In conclusion. In this study, aimed at investigating the economic and production effects of herds obtaining BVDV-free certification in the final stage of a national BVDV-free program, we did not see any economic or herd performance improvements in herds certified as BVDV free. When designing national programs to eradicate BVDV, it is important to include incentives for such farms to motivate them to join the program.

## Data Availability Statement

The data analyzed in this study is subject to the following licenses/restrictions: The datasets analyzed during the current study are not publicly available but are available from the author on reasonable request. Requests to access these datasets should be directed to HH, henk.hogeveen@wur.nl.

## Author Contributions

XY, JW, MV, WS, and HH: design of the study. XY, JW, and HH: data analysis. XY: drafting the manuscript. MV and WS: drafting the manuscript and critical revision of the article. HH: critical revision of the article. All authors contributed to the article and approved the submitted version.

## Funding

This research was funded by the China Scholarship Council (CSC, Beijing, China; Grant No. 201706350278) and the Sino-Dutch Dairy Development Center (SDDDC, Beijing, China).

## Conflict of Interest

The authors declare that the research was conducted in the absence of any commercial or financial relationships that could be construed as a potential conflict of interest.

## Publisher's Note

All claims expressed in this article are solely those of the authors and do not necessarily represent those of their affiliated organizations, or those of the publisher, the editors and the reviewers. Any product that may be evaluated in this article, or claim that may be made by its manufacturer, is not guaranteed or endorsed by the publisher.

## References

[1] Richter V Lebl K Baumgartner W Obritzhauser W Käsbohrer A Pinior B. A systematic worldwide review of the direct monetary losses in cattle due to bovine viral diarrhoea virus infection. Vet J. (2017) 220:80–7. 10.1016/j.tvjl.2017.01.005 28190502

[2] Pinior B Garcia S J Minviel J Raboisson D Minviel JJ Raboisson D. Epidemiological factors and mitigation measures influencing production losses in cattle due to bovine viral diarrhoea virus infection: a meta-analysis. Transbound Emerg Dis. (2019) 66:2426–39. 10.1111/tbed.13300 31328411 PMC6900039

[3] Grooms DL. Reproductive losses caused by bovine viral diarrhea virus and leptospirosis. Theriogenology. (2006) 66:624–8. 10.1016/j.theriogenology.2006.04.016 16716386

[4] Stott AW Humphry RW Gunn GJ. Modelling the effects of previous infection and re-infection on the costs of bovine viral diarrhoea outbreaks in beef herds. Vet J. (2010) 185:138–43. 10.1016/j.tvjl.2009.05.020 19709915

[5] Damman A Viet AF Arnoux S Guerrier-Chatellet MC Petit E Ezanno P. Modelling the spread of bovine viral diarrhea virus (BVDV) in a beef cattle herd and its impact on herd productivity. Vet Res. (2015) 46:12. 10.1186/s13567-015-0145-8 25828555 PMC4337316

[6] Moennig V Houe H Lindberg A. BVD control in Europe: current status and perspectives. Anim Heal Res Rev. (2007) 6:63–74. 10.1079/AHR2005102 16164009

[7] Thomann B Tschopp A Magouras I Meylan M Schüpbach-Regula G Häsler B. Economic evaluation of the eradication program for bovine viral diarrhea in the Swiss dairy sector. Prev Vet Med. (2017) 145:1–6. 10.1016/j.prevetmed.2017.05.020 28903865

[8] Joly A Fourichon C Beaudeau F. Description and first results of a BVDV control scheme in Brittany (western France). Prev Vet Med. (2005) 72:209–213. 10.1016/j.prevetmed.2005.07.016 16242196

[9] Graham DA Clegg TA O'sullivan P More SJ. Survival time of calves with positive BVD virus results born during the voluntary phase of the Irish eradication programme. Prev Vet Med. (2015) 119:123–33. 10.1016/j.prevetmed.2015.02.011 25769193

[10] Heffernan C Azbel-Jackson L Brownlie J Gunn G. Farmer attitudes and livestock disease: exploring citizenship behaviour and peer monitoring across two BVD control schemes in the UK. PLoS ONE. (2016) 11:e0152295. 10.1371/journal.pone.0152295 27023269 PMC4811405

[11] Kaiser V Nebel L Schüpbach-Regula G Zanoni RG Schweizer M. Influence of border disease virus (BDV) on serological surveillance within the bovine virus diarrhea (BVD) eradication program in Switzerland. BMC Vet Res. (2016) 13:1–13. 10.1186/s12917-016-0932-0 28086880 PMC5237232

[12] Wernike K Gethmann J Schirrmeier H Schröder R Conraths FJ Beer M. Six years (2011-2016) of mandatory nationwide bovine viral diarrhea control in Germany-a success story. (2017) 6:50. 10.3390/pathogens6040050 29057796 PMC5750574

[13] Mars MH Van Maanen C. Diagnostic assays applied in BVDV control in The Netherlands. Prev Vet Med. (2005) 72:43–8. 10.1016/j.prevetmed.2005.08.005 16199104

[14] GD Animal Health. Monitoring diergezondheid rundvee. Hoofdpunten Rapportage Derde kwartaal 2014. Deventer: GD (2014).

[15] Royal GD Veekijker Nieuws. (2019). Available online at: https://www.gddiergezondheid.nl/dapcontact/-/media/Files/Monitoringsflyers/Rund/22594GD2108Veekijkernieuwsrund20193WEBDEF.ashx (accessed June 30 2021)

[16] ZuivelNL. National Approach IBR and BVD Formally Started. (2018). Available online at: https://www.ibrbvd.nl/landelijke-aanpak-ibr-en-bvd-formeel-gestart/ (accessed June 15, 2021).

[17] Royal GD. Monitoring diergezondheid Rundvee, Hoofdpunten Eerste Kwartaal 2018. Deventer, NL: Royal GD (2018)

[18] Royal GD. Veekijker Nieuws. Deventer, NL: Royal GD (2021).

[19] Tschopp A Deiss R Rotzer M Wanda S Thomann B Schüpbach-Regula G. A matched case-control study comparing udder health, production and fertility parameters in dairy farms before and after the eradication of Bovine Virus Diarrhoea in Switzerland. Prev Vet Med. (2017) 144:29–39. 10.1016/j.prevetmed.2017.05.016 28716201

[20] Berends IMGA Swart WAJM Frankena K Muskens J Lam TJGM van Schaik G. The effect of becoming BVDV-free on fertility and udder health in dutch dairy herds. Prev Vet Med. (2008) 84:48–60. 10.1016/j.prevetmed.2007.11.002 18155307

[21] Burgstaller J Obritzhauser W Kuchling S Kopacka I Pinior B Kofer J. The effect of bovine viral diarrhoea virus on fertility in dairy cows: two case-control studies in the province of Styria, Austria. Berl Munch Tierarztl Wochenschr. (2016) 129:103–10. 10.2376/0005-9366-129-103 27169147

[22] Bitsch V Hansen K-EEL Rønsholt L. Experiences from the Danish programme for eradication of bovine virus diarrhoea (BVD) 1994–1998 with special reference to legislation and causes of infection. Vet Microbiol. (2000) 77:137–43. 10.1016/S0378-1135(00)00270-4 11042407

[23] Valle PS Skjerve E Martin SW Larssen RB Østerås O Nyberg O. Ten years of bovine virus diarrhoea virus (BVDV) control in norway: a cost-benefit analysis. Prev Vet Med. (2005) 72:189–207. 10.1016/j.prevetmed.2005.07.017 16213612

[24] Presi P Struchen R Knight-Jones T Scholl S Heim D. Bovine viral diarrhea (BVD) eradication in Switzerland–experiences of the first two years. Prev Vet Med. (2011) 99:112–21. 10.1016/j.prevetmed.2011.01.012 21371766

[25] Pinior B Firth CL Richter V Lebl K Trauffler M Dzieciol M. A systematic review of financial and economic assessments of bovine viral diarrhea virus (BVDV) prevention and mitigation activities worldwide. Prev Vet Med. (2017) 137:77–92. 10.1016/j.prevetmed.2016.12.014 28040270

[26] Dufour B Repiquet D Touratier A. Place des études économiques dans les décisions de santé animale : exemple du rapport coût/bénéfice de l'éradication de la diarrhée virale bovine en France. Rev Sci Tech Int des épizooties. (1999) 18:520–32. 10.20506/rst.18.2.1175 10472683

[27] Hasler B Howe KS Presi P Stark KDC. An economic model to evaluate the mitigation programme for bovine viral diarrhoea in Switzerland. Prev Vet Med. (2012) 106:162–73. 10.1016/j.prevetmed.2012.01.022 22402180

[28] Stott AW Humphry RW Gunn GJ Higgins I Hennessy T O'Flaherty J. Predicted costs and benefits of eradicating BVDV from Ireland. Ir Vet J. (2012) 65:1–11. 10.1186/2046-0481-65-12 22748235 PMC3443014

[29] Santman-Berends IMGA Mars MH van Duijn L van Schaik G. Evaluation of the epidemiological and economic consequences of control scenarios for bovine viral diarrhea virus in dairy herds. J Dairy Sci. (2015) 98:7699–716. 10.3168/jds.2014-9255 26364098

[30] R Core Team. R: A Language and Environment for Statistical Computing. Vienna: R Foundation for Statistical Computing (2018).

[31] D'Agostino RB Jr. Propensity score methods for bias reduction in the comparison of a treatment to a non-randomized control group. Stat Med. (1998) 17:2265–81. 10.1002/(SICI)1097-0258(19981015)17:19<2265::AID-SIM918>3.0.CO 9802183

[32] Rosenbaum PR Rubin DB. The central role of the propensity score in observational studies for causal effects. Biometrika. (1983) 70:41–55. 10.1093/biomet/70.1.41

[33] Caliendo M Kopeinig S. Some practical guidance for the implementation of propensity score matching. J Econ Surv. (2008) 22:31–72. 10.1111/j.1467-6419.2007.00527.x35387508

[34] Rose S Van Der Laan MJ. Why match? Investigating matched case-control study designs with causal effect estimation. Int J Biostat. (2009) 5. 10.2202/1557-4679.1127 PMC2827892 20231866

[35] Leuven E Sianesi B. PSMATCH2: Stata Module to Perform Full Mahalanobis Propensity Score Matching, Common Support Graphing, Covariate Imbalance Testing. (2003). Available online at: https://ideas.repec.org/c/boc/bocode/s432001.html (accessed May 25, 2021).

[36] StataCorp. Stata Statistical Software: Release 14. College Station, TX: StataCorp LP (2017).

[37] Rosenbaum PR Rubin DB. Constructing a control group using multivariate matched sampling methods that incorporate the propensity score. Am Stat. (1985) 39:33–8. 10.1080/00031305.1985.10479383

[38] Haviland A Nagin DS Rosenbaum PR. Combining propensity score matching and group-based trajectory analysis in an observational study. Psychol Meth. (2007) 12:247. 10.1037/1082-989X.12.3.247 17784793

[39] Austin PC. A comparison of 12 algorithms for matching on the propensity score. Stat Med. (2014) 33:1057–69. 10.1002/sim.6004 24123228 PMC4285163

[40] Ashenfelter O Card D. Using the longitudinal structure of earnings to estimate the effect of training programs. Rev Econ Stat. (1985) 67:648. 10.2307/1924810

[41] Beck T Levine R Levkov A. Big bad banks? The winners and losers from bank deregulation in the United States. J Financ. (2010) LXV:1637–67. 10.1111/j.1540-6261.2010.01589.x

[42] Callaway B Sant'Anna PHC. Difference-in-Differences with multiple time periods. J Econom. (2020) 225:200–30. 10.1016/j.jeconom.2020.12.001

[43] Lindberg A Emanuelson U. Effect of Bovine Viral Diarrhea Virus Infection on Average Annual Milk Yield and Average Bulk Milk Somatic Cell Counts in Swedish Dairy Herds. In Epidémiologie et Sante Animale. Paris: International Symposia on Veterinary Epidemiology and Economics.

[44] StataCorp. xtreg — Fixed-, Between-, and Random-Effects and Population-Averaged Linear Models. (2021). Available online at: https://www.stata.com/manuals/xtxtreg.pdf (accessed May 20. 2021).

[45] Wing C Simon K Bello-Gomez RA. Designing difference in difference studies: best practices for public health policy research. Annu Rev Public Health. (2018) 39:453–69. 10.1146/annurev-publhealth-040617-013507 29328877

[46] Stuart EA Huskamp HA Duckworth K Simmons J Song Z Chernew ME. Using propensity scores in difference-in-differences models to estimate the effects of a policy change. Heal Serv Outcomes Res Method. (2014) 14:166–82. 10.1007/s10742-014-0123-z 25530705 PMC4267761

[47] CRV. Farms and Cows in Numbers - the Netherlands. (2021). Available online at: https://www.cooperatie-crv.nl/downloads/stamboek/bedrijven-en-koeien-in-cijfers/ (accessed June 27, 2021).

[48] Bryson A Dorsett R Purdon S. The Use of Propensity Score Matching in the Evaluation of Active Labour Market Policies. London: Department for Work and Pensions (2002).

[49] Niskanen R Emanuelson U Sundberg J Larsson B Alenius S. Effects of infection with bovine virus diarrhoea virus on health and reproductive performance in 213 dairy herds in one county in Sweden. Prev Vet Med. (1995) 23:229–37. 10.1016/0167-5877(94)00437-N

[50] Beaudeau F Fourichon C Robert a Joly a Seegers H. Milk Yield of Cows and Bovine Viral Diarrhoea Virus (BVDV) Infection in 7,252 Dairy Herds in Bretagne (Western France). In 55th Annual Meeting of the European Association for Animal Production (EAAP). Bled: Wageningen Academic Publishers (2004).

[51] Fourichon C Beaudeau F Bareille N Seegers H. Quantification of economic losses consecutive to infection of a dairy herd with bovine viral diarrhoea virus. Prev Vet Med. (2005) 72:177–81. 10.1016/j.prevetmed.2005.08.018 16162364

[52] Knific T Zgajnar J. Modelling the Economic Impacts of Bovine Viral Diarrhoea Virus at Dairy Herd Level; The Case of Slovenia. Ljubljana: European Association of Agricultural Economists (EAAE) (2014).

[53] Houe H. Economic impact of BVDV infection in dairies. Biologicals. (2003) 31:137–43. 10.1016/S1045-1056(03)00030-7 12770546

[54] Evans CA Pinior B Larska M Graham D Schweizer M Guidarini C. Global knowledge gaps in the prevention and control of bovine viral diarrhoea (BVD) virus. Transbound Emerg Dis. (2019) 66:640–52. 10.1111/tbed.13068 30415496

[55] Brownlie J Mc C Cj H Dh P. Pathogenesis and epidemiology of bovine virus diarrhoea virus infection of cattle. Ann Rech Vet. (1987) 18:157–66. 3619343

[56] Baker JC. Clinical aspects of bovine virus diarrhoea virus infection. Rev Sci Tech. (1990) 9:25–41. 10.20506/rst.9.1.492 2132152

[57] Yue X Steeneveld W van der Voort M van Schaik G Vernooij JCM van Duijn L. The effect of bovine viral diarrhea virus introduction on milk production of dutch dairy herds. J Dairy Sci. (2021) 104:2074–86. 10.3168/jds.2020-18866 33309379

[58] Yue X van der Voort M Steeneveld W van Schaik G Vernooij JCM van Duijn L. The effect of new bovine viral diarrhea virus introduction on somatic cell count, calving interval, culling, and calf mortality of dairy herds in the dutch bovine viral diarrhea virus–free program. J Dairy Sci. (2021) 104:10217–31. 10.3168/jds.2021-20216 34147217

[59] Toplak I Hostnik P Cerne D Mrkun J Starič J. The principles of the voluntary programme for the control and elimination of bovine viral diarrhoea virus (BVDV) from infected herds in Slovenia. Front Vet Sci. (2021) 8. 10.3389/fvets.2021.676473 PMC8328193 34350227

[60] van Duijn L Veldhuis AMB Mars MH de Roo B Lam TJGM. Efficacy of a voluntary BVDV control programme: experiences from the Netherlands. Vet J. (2019) 245:55–60. 10.1016/j.tvjl.2018.12.016 30819426

[61] Vredenberg I Han R Mourits M Hogeveen H Steeneveld W. An empirical analysis on the longevity of dairy cows in relation to economic herd performance. Front Vet Sci. (2021) 8. 10.3389/fvets.2021.646672 PMC8071937 33912606

[62] Jongeneel R Daatselaar C van Leeuwen M Silvis H. Phosphate production reduction decree of the Netherlands: impact on markets, environment and dairy farm structure. Den Haag Wagen Eco Res. (2017). 10.18174/404867

[63] CRV. Jaarstatistieken-NL. www.crv.nl. (2018). Available online at: www.crv.nl (accessed June 25, 2021).

[64] Kulkarni P Mourits M Nielen M van den Broek J Steeneveld W. Survival analysis of dairy cows in the Netherlands under altering agricultural policy. Prev Vet Med. (2021) 193:105398. 10.1016/j.prevetmed.2021.105398 34119858

[65] Steeneveld W Hogeveen H Lansink AO. Economic consequences of investing in sensor systems on dairy farms. Comput Electron Agric. (2015) 119:33–9. 10.1016/j.compag.2015.10.006

