# Supplementary material for: Estimating the Effect of a Bovine Viral Diarrhea Virus Control Program: An Empirical Study on the Performance of Dutch Dairy Herds
Source: Front Vet Sci. 2022 Jul 7;9:892928. doi: 10.3389/fvets.2022.892928 (PMC9301250; doi:10.3389/fvets.2022.892928)
Supplement: Supplementary file 1 [file Data_Sheet_1.docx]

Estimating the Effect of a Bovine Viral Diarrhea Virus Control Program: An Empirical Study on the Performance of Dutch Dairy Herds

# SUPPLMENTARY MATERIALS

**Table S1.** Comparison of mean value, % bias^1^, t-value, and p-value of the matching covariates for the case^2^ and control herds^3^ before and after propensity score matching for each sub-data set for the analysis of calving interval and gross margin in the first analysis

| Sub-data set for the first analysis | Variables | Unmatched/  Matched | Mean | | %bias | t | $p$-value |
| --- | --- | --- | --- | --- | --- | --- | --- |
|  |  |  | Case herds | Control herds |  |  |  |
| Case herds changed BVDV status in 2015 | Farm intensity, n/ha | Unmatched | 2.31 | 2.18 | 19.4 | 0.37 | 0.715 |
|  |  | Matched | 1.88 | 2.01 | -19.4 | -0.46 | 0.688 |
|  | Herd size | Unmatched | 126 | 117 | 23.5 | 0.32 | 0.749 |
|  |  | Matched | 135 | 118 | 45.9 | 0.35 | 0.763 |
|  | Milk yield, kg/cow/year | Unmatched | 7214 | 8720 | -213.2 | -3.06 | 0.003 |
|  |  | Matched | 7492 | 7452 | 5.7 | 0.08 | 0.944 |
| Case herds changed BVDV status in 2016 | Farm intensity, n/ha | Unmatched | 2.48 | 2.22 | 38.7 | 0.81 | 0.419 |
|  |  | Matched | 2.65 | 2.32 | 47.2 | 0.52 | 0.631 |
|  | Herd size | Unmatched | 105 | 126 | -48.8 | -0.76 | 0.449 |
|  |  | Matched | 114 | 113 | 2.7 | 0.04 | 0.971 |
|  | Milk yield, kg/cow/year | Unmatched | 7900 | 8853 | -113.4 | -2.23 | 0.028 |
|  |  | Matched | 8306 | 8033 | 32.5 | 0.6 | 0.582 |
| Case herds changed BVDV status in 2017 | Farm intensity, n/ha | Unmatched | 2.11 | 2.07 | 8.0 | 0.24 | 0.811 |
|  |  | Matched | 1.92 | 2.05 | -22.8 | -0.58 | 0.572 |
|  | Herd size | Unmatched | 100 | 127 | -57.2 | -1.32 | 0.189 |
|  |  | Matched | 102 | 108 | -13.4 | -0.34 | 0.737 |
|  | Milk yield, kg/cow/year | Unmatched | 8727 | 9050 | -26.0 | -0.91 | 0.363 |
|  |  | Matched | 9256 | 9089 | 13.5 | 0.48 | 0.640 |
| Case herds changed BVDV status in 2018 | Farm intensity, n/ha | Unmatched | 2.10 | 2.05 | 14.6 | 0.18 | 0.861 |
|  |  | Matched | 1.88 | 2.09 | -53.0 | . | . |
|  | Herd size | Unmatched | 208 | 123 | 62.9 | 2.08 | 0.041 |
|  |  | Matched | 79 | 118 | -29.1 | . | . |
|  | Milk yield, kg/cow/year | Unmatched | 9237 | 9150 | 14.0 | 0.14 | 0.886 |
|  |  | Matched | 9082 | 9293 | -33.9 | . | . |
| Case herds changed BVDV status in 2019 | Farm intensity, n/ha | Unmatched | 2.46 | 2.07 | 118.7 | 1.18 | 0.073 |
|  |  | Matched | 2.46 | 2.41 | 13.2 | 0.35 | 0.739 |
|  | Herd size | Unmatched | 148 | 128 | 34.8 | 0.66 | 0.512 |
|  |  | Matched | 148 | 138 | 17.1 | 0.21 | 0.838 |
|  | Milk yield, kg/cow/year | Unmatched | 9004 | 9195 | 30.1 | -0.45 | 0.651 |
|  |  | Matched | 9004 | 8802 | 30.9 | 0.35 | 0.736 |

^1^ % bias = the standardized percentage bias, which is the percentage difference of the sample means in the case and control sub-samples as a percentage of the square root of the average of the sample variances in the case and control herds (Leuven; Rosenbaum and Rubin, 1985).

^2^ Case herds = herds of which the BVDV status changed from ‘BVDV-not-free’ to ‘BVDV-free’ during the study period. The not-free status refers to a herd that participated in the BVDV-free program but had not yet obtained the BVDV-free certification.

^3^ Control herds = herds that were BVDV-free during the entire study period.

**Table S2.** Comparison of mean value, % bias^1^, t-value, and p-value of the matching covariates for the case^2^ and control herd^3^ before and after propensity score matching for each sub-data set for the analysis of milk yield and somatic cell count in the first analysis

| Sub-data set for the second analysis | Variables | Unmatched/Matched | Mean | | %bias^3^ | t | $p$-value |
| --- | --- | --- | --- | --- | --- | --- | --- |
|  |  |  | Case herds^1^ | Control herds^2^ |  |  |  |
| Case herds changed BVDV status in 2015 | Farm intensity, n/ha | Unmatched | 2.31 | 2.18 | 19.4 | 0.37 | 0.715 |
|  |  | Matched | 2.31 | 2.18 | 1.2 | 0.02 | 0.988 |
|  | Herd size | Unmatched | 126 | 117 | 23.5 | 0.32 | 0.749 |
|  |  | Matched | 126 | 121 | 11.9 | 0.18 | 0.864 |
| Case herds changed BVDV status in 2016 | Farm intensity, n/ha | Unmatched | 2.48 | 2.22 | 38.7 | 0.81 | 0.419 |
|  |  | Matched | 2.48 | 2.40 | 11.8 | 0.17 | 0.868 |
|  | Herd size | Unmatched | 105 | 126 | -48.8 | -0.76 | 0.449 |
|  |  | Matched | 105 | 102 | 7.6 | 0.16 | 0.875 |
| Case herds changed BVDV status in 2017 | Farm intensity, n/ha | Unmatched | 2.11 | 2.07 | 8 | 0.24 | 0.811 |
|  |  | Matched | 2.11 | 2.09 | 3.9 | 0.07 | 0.942 |
|  | Herd size | Unmatched | 100 | 127 | -57.2 | -1.32 | 0.189 |
|  |  | Matched | 100 | 101 | -3.2 | -0.09 | 0.929 |
| Case herds changed BVDV status in 2018 | Farm intensity, n/ha | Unmatched | 2.10 | 2.05 | 14.6 | 0.18 | 0.861 |
|  |  | Matched | 1.88 | 1.88 | 1 | . | . |
|  | Herd size | Unmatched | 208 | 123 | 62.9 | 2.08 | 0.041 |
|  |  | Matched | 79 | 81 | -1.5 | . | . |
| Case herds changed BVDV status in 2019 | Farm intensity, n/ha | Unmatched | 2.46 | 2.07 | 118.7 | 1.81 | 0.073 |
|  |  | Matched | 2.46 | 2.45 | 2.6 | 0.07 | 0.948 |
|  | Herd size | Unmatched | 148 | 128 | 34.8 | 0.66 | 0.512 |
|  |  | Matched | 148 | 147 | 1.3 | 0.02 | 0.987 |

^1^ % bias = the standardized percentage bias, which is the percentage difference of the sample means in the case and control sub-samples as a percentage of the square root of the average of the sample variances in the case and control herds (Leuven; Rosenbaum and Rubin, 1985).

^2^ Case herds = herds of which the BVDV status changed from ‘BVDV-not-free’ to ‘BVDV-free’ during the study period. The not-free status refers to a herd that participated in the BVDV-free program but had not yet obtained the BVDV-free certification.

^3^ Control herds = herds that were BVDV-free during the entire study period

**Table S3.** Comparison of mean value, % bias^1^, t-value, and p-value of the matching covariates for the case^2^ and control herds^3^ before and after propensity score matching for each sub-data set for the analysis of calving interval and gross margin in the second analysis

| Sub-data set for the second analysis | Variables | Unmatched/  Matched | Mean | | %bias | t | $p$-value |
| --- | --- | --- | --- | --- | --- | --- | --- |
|  |  |  | Case herds | Control herds |  |  |  |
| Case herds changed BVDV status in 2015 | Farm intensity, n/ha | Unmatched | 1.91 | 2.18 | −54.7 | −1.13 | 0.260 |
|  |  | Matched | 1.91 | 1.91 | 0.3 | 0.01 | 0.992 |
|  | Herd size | Unmatched | 103 | 117 | −34.6 | −0.74 | 0.463 |
|  |  | Matched | 103 | 100 | 9.7 | 0.27 | 0.789 |
|  | Milk yield, kg/cow/year | Unmatched | 8918 | 8720 | 30.5 | 0.61 | 0.542 |
|  |  | Matched | 8918 | 8824 | 14.4 | 0.21 | 0.837 |
| Case herds changed BVDV status in 2016 | Farm intensity, n/ha | Unmatched | 1.91 | 2.22 | −58.9 | −1.74 | 0.085 |
|  |  | Matched | 1.96 | 1.91 | 10.6 | 0.39 | 0.700 |
|  | Herd size | Unmatched | 115 | 126 | −22.8 | −0.74 | 0.462 |
|  |  | Matched | 113 | 111 | 2.9 | 0.08 | 0.940 |
|  | Milk yield, kg/cow/year | Unmatched | 8419 | 8853 | −52.4 | −1.82 | 0.072 |
|  |  | Matched | 8618 | 8731 | −13.6 | −0.38 | 0.711 |
| Case herds changed BVDV status in 2017 | Farm intensity, n/ha | Unmatched | 2.22 | 2.07 | 27 | 0.98 | 0.327 |
|  |  | Matched | 2.07 | 2.08 | −2.1 | −0.07 | 0.944 |
|  | Herd size | Unmatched | 112 | 127 | −30.9 | −0.91 | 0.366 |
|  |  | Matched | 115 | 115 | 0.1 | 0.00 | 0.998 |
|  | Milk yield, kg/cow/year | Unmatched | 8776 | 9050 | −23.6 | −0.96 | 0.341 |
|  |  | Matched | 9051 | 9037 | 1.2 | 0.04 | 0.972 |
| Case herds changed BVDV status in 2018 | Farm intensity, n/ha | Unmatched | 2.47 | 2.05 | 50.2 | 2.07 | 0.041 |
|  |  | Matched | 2.09 | 2.09 | −0.8 | −0.03 | 0.981 |
|  | Herd size | Unmatched | 103 | 123 | −50.2 | −0.98 | 0.329 |
|  |  | Matched | 98 | 98 | −0.5 | −0.01 | 0.990 |
|  | Milk yield, kg/cow/year | Unmatched | 9305 | 9150 | 20.8 | 0.47 | 0.639 |
|  |  | Matched | 9157 | 9180 | −3.1 | −0.05 | 0.961 |
| Case herds changed BVDV status in 2019 | Farm intensity, n/ha | Unmatched | 2.22 | 2.07 | 37.6 | 0.68 | 0.497 |
|  |  | Matched | 2.22 | 2.23 | −3.5 | −0.05 | 0.964 |
|  | Herd size | Unmatched | 135 | 128 | 9.3 | 0.22 | 0.828 |
|  |  | Matched | 135 | 138 | −3.8 | −0.05 | 0.963 |
|  | Milk yield, kg/cow/year | Unmatched | 8582 | 9195 | −76 | −1.44 | 0.153 |
|  |  | Matched | 8582 | 8621 | −4.9 | −0.08 | 0.941 |

^1^ % bias = the standardized percentage bias, which is the percentage difference of the sample means in the case and control sub-samples as a percentage of the square root of the average of the sample variances in the case and control herds (Leuven; Rosenbaum and Rubin, 1985).

^2^ Case herds = herds that started participating in the Dutch BVDV-free program during the study period and obtained the BVDV-free certification.

^3^ Control herds = herds that were BVDV-free during the entire study period.

**Table S4.** Comparison of mean value, % bias^1^, t-value, and p-value of the matching covariates for the case^2^ and control herd^3^ before and after propensity score matching for each sub-data set for the analysis of milk yield and somatic cell count in the second analysis

| Sub-data set for the second analysis | Variables | Unmatched/Matched | Mean | | %bias^3^ | t | $p$-value |
| --- | --- | --- | --- | --- | --- | --- | --- |
|  |  |  | Case herds^1^ | Control herds^2^ |  |  |  |
| Case herds changed BVDV status in 2015 | Farm intensity, n/ha | Unmatched | 1.91 | 2.18 | −54.7 | −1.13 | 0.260 |
|  |  | Matched | 1.91 | 1.92 | −1.9 | −0.06 | 0.950 |
|  | Herd size | Unmatched | 103 | 117 | −34.6 | −0.74 | 0.463 |
|  |  | Matched | 103 | 103 | 0.3 | 0.01 | 0.995 |
| Case herds changed BVDV status in 2016 | Farm intensity, n/ha | Unmatched | 1.91 | 2.22 | −58.9 | −1.74 | 0.085 |
|  |  | Matched | 1.91 | 1.92 | −1.7 | −0.07 | 0.944 |
|  | Herd size | Unmatched | 115 | 126 | −22.8 | −0.74 | 0.462 |
|  |  | Matched | 115 | 115 | −1.2 | −0.03 | 0.974 |
| Case herds changed BVDV status in 2017 | Farm intensity, n/ha | Unmatched | 2.22 | 2.07 | 27 | 0.98 | 0.327 |
|  |  | Matched | 2.07 | 2.05 | 3.3 | 0.12 | 0.906 |
|  | Herd size | Unmatched | 112 | 127 | −30.9 | −0.91 | 0.366 |
|  |  | Matched | 115 | 111 | 8.9 | 0.29 | 0.775 |
| Case herds changed BVDV status in 2018 | Farm intensity, n/ha | Unmatched | 2.47 | 2.05 | 50.2 | 2.07 | 0.041 |
|  |  | Matched | 2.09 | 2.13 | −4.7 | −0.14 | 0.888 |
|  | Herd size | Unmatched | 103 | 123 | −50.2 | −0.98 | 0.329 |
|  |  | Matched | 98 | 98 | 0.4 | 0.01 | 0.991 |
| Case herds changed BVDV status in 2019 | Farm intensity, n/ha | Unmatched | 2.22 | 2.07 | 37.6 | 0.68 | 0.497 |
|  |  | Matched | 2.22 | 2.21 | 2.6 | 0.04 | 0.968 |
|  | Herd size | Unmatched | 135 | 128 | 9.3 | 0.22 | 0.828 |
|  |  | Matched | 135 | 128 | 9.2 | 0.13 | 0.900 |

^1^ % bias = the standardized percentage bias, which is the percentage difference of the sample means in the case and control sub-samples as a percentage of the square root of the average of the sample variances in the case and control herds (Leuven; Rosenbaum and Rubin, 1985).

^2^ Case herds = herds that started participating in the Dutch BVDV-free program during the study period and obtained the BVDV-free certification.

^3^ Control herds = herds that were BVDV-free during the entire study period.

**Table S5A.** The descriptive statistics of the performance in the studied Dutch dairy herds from 2014 to 2019 in the first analysis^1^

| Variable | Data set 1.1 (first analysis of calving interval and gross margin, case herds changed from ‘BVDV not free’ to ‘BVDV free’) | | | | | | | |
| --- | --- | --- | --- | --- | --- | --- | --- | --- |
|  | Case herds | | | Control herds | | | | |
|  | Annual BVDV Status | | | Years matched to case herds | | | | |
|  | Year -1  (not free) | Year 0  (free) | Year 1  (free) | Year -1  (free) | Year 0  (free) | | Year 1  (free) | |
| Herd size, n cows | 118 (43) | 117 (41) | 107 (37) | 120 (40) | 118 (41) | | 116 (39) | |
| Land use, ha | 55 (18) | 55 (17) | 55 (16) | 56 (19) | 56 (19) | | 57 (18) | |
| Farm intensity,  n cows/ha/year | 2.18 (0.56) | 2.17 (0.47) | 1.90 (0.29) | 2.23 (0.58) | 2.14 (0.51) | | 2.10 (0.49) | |
| Milk yield,  kg/cow/year | 8,640  (507) | 8,810  (647) | 8,830  (919) | 8,830  (911) | 8,990  (931) | | 9,080  (896) | |
| Fat, % | 4.36 (0.12) | 4.38 (0.14) | 4.38 (0.16) | 4.41 (0.16) | 4.41 (0.16) | | 4.42 (0.17) | |
| Protein, % | 3.52 (0.08) | 3.55 (0.11) | 3.52 (0.08) | 3.53 (0.09) | 3.54 (0.09) | | 3.55 (0.09) | |
| SCC,  1,000 cells/mL | 176 (79) | 170 (60) | 168 (66) | 168 (55) | 163 (53) | | 156 (53) | |
| Calving interval,  days | 406 (23) | 404 (18) | 413 (29) | 407 (22) | 406 (19) | | 404 (17) | |
| Non-return rate, % | 59 (14) | 63 (15) | 69 (15) | 58 (12) | 58 (13) | | 59 (13) | |
| Inseminations, n | 2.15 (0.40) | 2.12 (0.50) | 1.86 (0.40) | 2.18 (0.42) | 2.18 (0.46) | | 2.18 (0.49) | |
| Milk return,  euro/kg milk | 0.350 (0.041) | 0.371  (0.044) | 0.369 (0.045) | 0.349  (0.046) | 0.378  (0.037) | | 0.385 (0.024) | |
| Gross margin,  euro/kg milk | 0.238 (0.052) | 0.264  (0.056) | 0.276 (0.053) | 0.260  (0.055) | 0.289  (0.051) | | 0.296 (0.044) | |
| Variable | Data set 2.1 (first analysis of milk yield and SCC, case herds changed from ‘BVDV not free’ to ‘BVDV free’) | | | | | | | |
|  | Case herds | | | Control herds | | | | |
|  | Annual BVDV Status | | | Years matched to case herds | | | | |
|  | Year -1  (not free) | Year 0  (free) | Year 1  (free) | Year -1  (free) | | Year 0 (free) | | Year 1  (free) |
| Herd size, n cows | 112 (40) | 116 (41) | 103 (32) | 113 (34) | | 113 (35) | | 112 (36) |
| Land use, ha | 51 (17) | 52 (20) | 50 (18) | 53 (18) | | 53 (17) | | 54 (19) |
| Farm intensity,  n cows/ha/year | 2.26 (0.61) | 2.30 (0.57) | 2.13 (0.53) | 2.20 (0.50) | | 2.19 (0.47) | | 2.13 (0.46) |
| Milk yield, kg/cow/year | 8,270  (1,240) | 8,320  (1,240) | 8,230 (1,450) | 8,860  (830) | | 8,990  (888) | | 9,060  (885) |
| Fat, % | 4.38 (0.13) | 4.36 (0.14) | 4.38 (0.13) | 4.39 (0.17) | | 4.40 (0.17) | | 4.41 (0.15) |
| Protein, % | 3.54 (0.09) | 3.53 (0.10) | 3.54 (0.09) | 3.51 (0.09) | | 3.53 (0.09) | | 3.52 (0.08) |
| SCC,  1,000 cells/mL | 193 (80) | 170 (58) | 175 (61) | 171 (52) | | 161 (47) | | 156 (48) |
| Calving interval,  days | 402 (23) | 408 (15) | 409 (26) | 407 (22) | | 407 (20) | | 404 (19) |
| Non-return rate, % | 63 (16) | 63 (16) | 69 (14) | 58 (13) | | 58 (13) | | 59 (13) |
| Inseminations, n | 2.01 (0.45) | 2.09 (0.54) | 1.84 (0.38) | 2.20 (0.45) | | 2.19 (0.48) | | 2.17 (0.47) |
| Milk return,  euro/kg milk | 0.360 (0.050) | 0.359  (0.045) | 0.360 (0.042) | 0.351  (0.050) | | 0.373 (0.036) | | 0.351 (0.042) |
| Gross margin,  euro/kg milk | 0.259 (0.058) | 0.258  (0.065) | 0.267 (0.057) | 0.261  (0.059) | | 0.282 (0.049) | | 0.264 (0.055) |

**Table S5B.** The descriptive statistics of the performance in the studied Dutch dairy herds from 2014 to 2019 in the second analysis^2^

| Variable | Data set 1.2 (second analysis of calving interval and gross margin, case herds changed from ‘not participating’ to ‘BVDV free’) | | | | | | | |
| --- | --- | --- | --- | --- | --- | --- | --- | --- |
|  | Case herds | | | Control herds | | | | |
|  | Annual BVDV Status | | | Years matched to case herds | | | | |
|  | Year -1  (not participating) | Year 0  (free) | Year 1  (free) | Year -1  (free) | | Year 0  (free) | | Year 1  (free) |
| Herd size, n cows | 110 (40) | 112 (40) | 107 (34) | 118 (45) | | 118 (46) | | 111 (40) |
| Land use, ha | 55 (20) | 56 (20) | 56 (17) | 56 (21) | | 57 (21) | | 57 (21) |
| Farm intensity,  n cows/ha/year | 2.05 (0.46) | 2.03 (0.33) | 1.95 (0.29) | 2.15 (0.48) | | 2.09 (0.43) | | 1.99 (0.40) |
| Milk yield,  kg/cow/year | 8,740  (729) | 8,870  (744) | 9,040  (829) | 8,780  (819) | | 8,920  (845) | | 9,080  (890) |
| Fat, % | 4.41 (0.18) | 4.44 (0.17) | 4.41 (0.21) | 4.4 (0.16) | | 4.41 (0.17) | | 4.41 (0.16) |
| Protein, % | 3.54 (0.10) | 3.56 (0.10) | 3.56 (0.09) | 3.52 (0.09) | | 3.54 (0.09) | | 3.53 (0.09) |
| SCC,  1,000 cells/mL | 164 (51) | 156 (43) | 153 (43) | 169 (51) | | 166 (52) | | 154 (48) |
| Calving interval,  days | 403 (20) | 403 (18) | 399 (15) | 405 (21) | | 406 (21) | | 406 (22) |
| Non-return rate, % | 62 (13) | 62 (13) | 61 (14) | 59 (13) | | 59 (13) | | 59 (13) |
| Inseminations, n | 2.06 (0.48) | 2.09 (0.48) | 2.04 (0.40) | 2.18 (0.49) | | 2.22 (0.51) | | 2.16 (0.48) |
| Milk return,  euro/kg milk | 0.359  (0.047) | 0.365 (0.050) | 0.385 (0.049) | 0.344 (0.044) | | 0.38 (0.035) | | 0.379  (0.030) |
| Gross margin,  euro/kg milk | 0.286  (0.058) | 0.288 (0.058) | 0.308 (0.052) | 0.256 (0.055) | | 0.291 (0.049) | | 0.292  (0.044) |
| Variable | Data set 2.2 (second analysis of milk yield and SCC, case herds changed from ‘not participating’ to ‘BVDV free’) | | | | | | | |
|  | Case herds | | | Control herds | | | | |
|  | Annual BVDV Status | | | Years matched to case herds | | | | |
|  | Year -1  (not participating) | Year 0  (free) | Year 1  (free) | Year -1 (free) | Year 0 (free) | | Year 1  (free) | |
| Herd size, n cows | 110 (40) | 113 (40) | 108 (34) | 108 (35) | 110 (38) | | 110 (40) | |
| Land use, ha | 57 (22) | 57 (20) | 58 (21) | 53 (17) | 55 (19) | | 56 (21) | |
| Farm intensity,  n cows/ha/year | 2.01 (0.48) | 2.01 (0.34) | 1.92 (0.31) | 2.07 (0.43) | 2.03 (0.36) | | 1.99 (0.37) | |
| Milk yield, kg/cow/year | 8,670  (784) | 8,790 (808) | 8,970  (851) | 8,780  (876) | 8,910 (866) | | 9,000  (866) | |
| Fat, % | 4.41 (0.18) | 4.44 (0.17) | 4.42 (0.21) | 4.37 (0.16) | 4.39 (0.15) | | 4.40 (0.16) | |
| Protein, % | 3.53 (0.11) | 3.56 (0.10) | 3.55 (0.09) | 3.51 (0.08) | 3.52 (0.08) | | 3.53 (0.08) | |
| SCC, 1,000 cells/mL | 162 (50) | 154 (43) | 150 (43) | 167 (52) | 161 (50) | | 158 (50) | |
| Calving interval, days | 402 (20) | 402 (18) | 398 (15) | 409 (23) | 407 (22) | | 406 (21) | |
| Non-return rate, % | 62 (12) | 62 (13) | 61 (14) | 59 (12) | 58 (12) | | 59 (12) | |
| Inseminations,  n | 2.05 (0.48) | 2.08 (0.48) | 2.03 (0.40) | 2.18 (0.41) | 2.18 (0.42) | | 2.18 (0.44) | |
| Milk return, euro/kg milk/year | 0.357  (0.047) | 0.362 (0.051) | 0.386 (0.048) | 0.358 (0.047) | 0.356 (0.041) | | 0.369  (0.039) | |
| Gross margin, euro/kg milk | 0.284  (0.057) | 0.286 (0.058) | 0.311 (0.054) | 0.271 (0.056) | 0.269 (0.050) | | 0.282  (0.050) | |

^1^ First analysis = case herds changed from ‘BVDV not free’ to ‘BVDV free’. The not-free status refers to a herd that participated in the BVDV-free program but had not yet obtained the BVDV-free certification.

^2^ Second analysis = case herds changed from ‘not participating’ to ‘BVDV free’.
